# Impact of Insect-Resistant Transgenic Maize 2A-7 on Diversity and Dynamics of Bacterial Communities in Rhizosphere Soil

**DOI:** 10.3390/plants12102046

**Published:** 2023-05-21

**Authors:** Xiaohui Xu, Xin Liu, Fan Li, Chaofeng Hao, Hongwei Sun, Shuke Yang, Yue Jiao, Xingbo Lu

**Affiliations:** 1 Shandong Key Laboratory of Plant Virology, Institute of Plant Protection, Shandong Academy of Agricultural Sciences, Jinan 250100, China; xuxiaohui1023@163.com (X.X.); liu18753136362@163.com (X.L.); lfzjnd@163.com (F.L.); chaofenghao2015@163.com (C.H.); hongweisun@126.com (H.S.); yangshuke426@126.com (S.Y.); 2 College of Life Sciences, Shandong Normal University, Jinan 250014, China; 3 Development Center for Science and Technology, Ministry of Agriculture and Rural Affairs, Beijing 100176, China; 4 Key Laboratory for Safety Assessment (Environment) of Agricultural Genetically Modified Organisms, Ministry of Agriculture and Rural Affairs, Jinan 250100, China

**Keywords:** insect-resistant transgenic maize, artificially modified Bt protein, bacterial community diversity, rhizosphere soil, risk assessment

## Abstract

Artificial modification of *Bacillus thuringiensis* (Bt) proteins can effectively improve their resistance to target pests, but the effect of such modification on the diversity of rhizosphere microorganisms remains unclear. Transgenic maize 2A-7 contains two artificially modified Bt proteins, mCry1Ab and mCry2Ab. These proteins can enter soil and pose a potential threat to soil microbial diversity. To assess their impacts on rhizosphere bacteria communities, the contents of the two Bt proteins and changes in bacterial community diversity in the rhizosphere soils of transgenic maize 2A-7 and its control variety were analyzed at different growth stages in 2020. The results showed that the two Bt proteins were detected at low levels in the rhizosphere soils of 2A-7 plants. No significant differences in soil bacterial diversity were detected between 2A-7 and its control variety at any of the growth stages. Bioinformatics analysis indicated that the growth stage, rather than the cultivar, was the main factor causing changes in bacterial communities. This research provides valuable data for understanding the impact of Bt crops on the soil microbiome, and establishes a theoretical basis for evaluation of their safety.

## 1. Introduction

Since the approval of genetically modified (GM) crops for commercialization in the United States in 1996, the global area of GM crops has expanded by more than 110 times, reaching 190.4 million hectares in 2019 [1]. GM crops have been planted or imported in more than 70 countries [2]. The rapid adoption of GM crops has brought enormous economic, social, and environmental benefits [3]. Currently, approximately 50% of commercialized GM crops contain insect-resistant traits. These insect-resistant GM crops can produce at least one *Bacillus thuringiensis* (Bt) toxin protein, which can directly kill target pests without the need for pesticide spraying throughout the whole growth period. The application of insect-resistant GM crops can not only improve the yield and safety of crops, but it can also save on labor input, promote the development of low-or no-tillage systems, and reduce greenhouse gas emissions. Thus, it is beneficial for the sustainable development of society and for the environment [4,5].

Previous studies have shown that the natural Bt toxin produced by *Bacillus thuringiensis* has no adverse impact on the environment [6]. GM crops expressing artificially modified Bt toxin proteins have strong toxicity and a broad insect resistance spectrum. In general, they target insects belong to the Lepidoptera, Diptera, and Coleoptera families; however, they also exhibit a certain level of toxicity to some non-target organisms, such as peach aphids [7]. During different growth stages, and after returning straw to the field, Bt proteins in GM crops enter the soil through exudates, pollen, and residues, which can be adsorbed by active particles in the soil and remain in the soil for a long time [8,9]. In soil, most Bt proteins form complexes with other soil factors, and their content levels are closely related to soil conditions such as type, temperature, pH, water content, and active particles [10]. During the adsorption process, protein concentration in soil usually exhibits a trend of first increasing, then decreasing, and finally reaching a steady state [11]. The content levels of Bt proteins and the activities of microbial communities are both elevated in rhizosphere soil compared with bulk soil. For this reason, changes in the structure and diversity of the rhizosphere microorganisms are often used as an early and effective indicators for assessing the effects of GM crops on soil ecology [12,13,14].

In the past ten years, a number of studies have been carried out to assess the biosafety of Bt crops with respect to soil microbial communities; however, their conclusions have been controversial. Two studies found that the planting of two Bt maize crops (Bt11 and Mon810) significantly altered the compositon, diversity and richness of fungal communities in rhizosphere soils [15,16]. In addition, transgenic cotton expressing the Cry1Ac protein was found to increase the functional diversity of soil microbial communities [17]. However, most Bt crops have been found to have no significant effects on soil microbial communities; these include Cry1Ac transgenic eggplant [18], transgenic maize Mon810 [19], Bt rice lines Huachi B6 and TT51 [20], and Cry3Bb maize MON863 [21]. Thus, studies on the impacts of GM crops on the soil ecosystem should fully consider the specificity of each transgenic plant, and be carried out on a “case-by-case” basis.

Various methods have been adopted for assessing the impact of GM crops on soil microbial diversity, such as plate colony-counting, Biolog, denaturing gradient gel electrophoresis (DGGE), and high-throughput sequencing [22,23,24]. The first three methods have obvious limitations, including high workloads, heavy time consumption and an inability to comprehensively reflect the composition, structure, and abundance of the microbial communities in the samples [25,26]. With the rapid development of high-throughput sequencing technology, this method is now widely used in environmental microbial diversity analyses [27,28]. Compared with traditional methods, high-throughput sequencing has a number of advantages, including low detection limits, high sensitivity, the absence of any culturing requirements, and the ability to systematically and rapidly evaluate the composition of samples (including known and unknown species), as well as structural variations in microbial communities. The content levels of microorganisms in rhizosphere soil are several times or even dozens of times higher than those in root-zone soil; among these microorganisms, bacteria, especially Gram-negative bacteria, are the most prevalent of all. Therefore, evaluating changes in the structure and diversity of the bacterial communities in rhizosphere soils can reflect, to a great extent, changes in microbial communities. The relative molecular weight of 16S rDNA is moderate, and the probability of its mutation is low. It consists of ten conservative regions and nine hypervariable regions [29]. Sequencing analysis of the hypervariable regions can easily distinguish different bacterial species. Therefore, 16S rDNA sequencing is widely used to evaluate microbial phylogeny, classification, and diversity [30,31,32].

In this study, to clarify the impact of transgenic maize 2A-7 expressing mCry1Ab and mCry2Ab genes on soil bacterial communities, the content levels of the two Bt proteins were measured in the rhizosphere soil of transgenic maize 2A-7 at different development stages in the summer of 2020. Then, the abundance, composition, and diversity of bacterial communities in the rhizosphere soil of transgenic maize 2A-7 and its control varieties were analyzed using 16s rDNA sequencing. A correlation analysis was conducted between changes in the bacterial community and changes in Bt protein content levels and the physicochemical properties [33] of rhizosphere soils, and the main factors leading to changes in bacterial communities were then identified. The results from the one-year field trial can provide necessary information for analyzing the impact of transgenic insect-resistant maize on soil bacterial communities, and provide a theoretical foundation for the biosafety regulation and commercialization of transgenic maize 2A-7.

## 2. Results

### 2.1. Changes of Bt Protein Contents in Rhizosphere Soil of Transgenic Maize 2A-7

**Figure 1:**
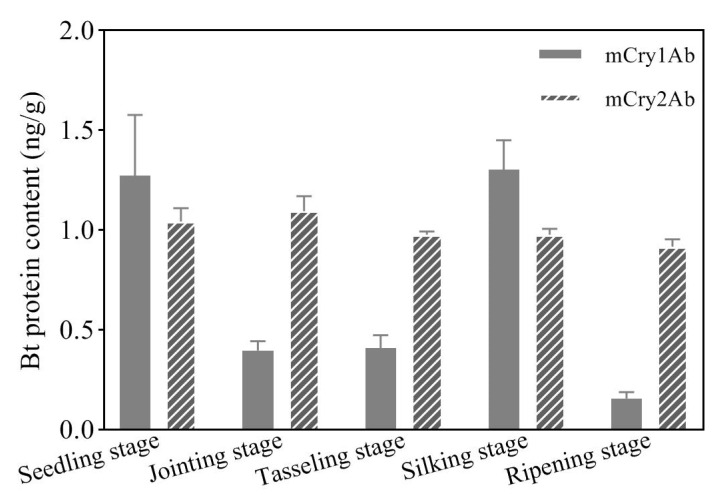
Content levels of mCry1Ab and mCry2Ab proteins in the rhizosphere soil of transgenic maize 2A-7 at different growth stages. Data are presented as the means ± standard deviations of three biological replicates.

To investigate the accumulation characteristics of the two modified Bt proteins (mCry1Ab and mCry2Ab) in the rhizosphere soil of transgenic maize 2A-7, the content levels of the two Bt proteins were measured with Cry1Ab and Cry2A Elisa kits, respectively. As shown in Figure 1, the two Bt proteins were detected in the rhizosphere soil of transgenic maize 2A-7 at relatively low levels, ranging from 0.15 ng/g to 1.16 ng/g (Figure 1). These values were less than one-thousandth of those of the two proteins expressed in the plant. Compared with the mCry2Ab protein, the content of mCry1Ab protein fluctuated greatly among different growth stages. The mCry1Ab content was highest at the seedling and silking stages, and lowest at the full ripening stage (Figure 1). The content of mCry2Ab was relatively stable at each growth stage, showing a slightly decreasing trend as the developmental process proceeded (Figure 1).

### 2.2. Alpha Diversity of Rhizosphere Bacterial Community of Transgenic Maize 2A-7 and Its Control

**Figure 2:**
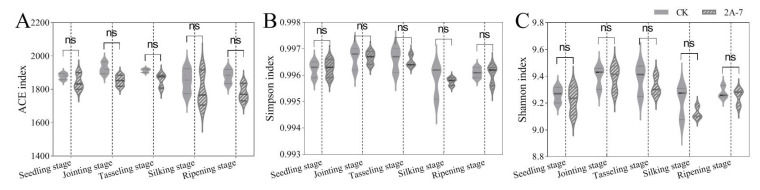
Comparison of three alpha diversity indices of rhizosphere bacterial communities from transgenic maize 2A-7 and its control. (**A**) ACE index; (**B**) Simpson index; (**C**) Shannon index. Significant difference analyses were carried out using a one way *t*-test and ANOVA. “ns” indicates no significant difference (*p* > 0.05). CK, non-transgenic control Dongdan 6531.

Alpha diversity reflects the richness and diversity of species in microbial communities. In the current study, three indices (ACE, Simpson and Shannon) were used to identify differences in the alpha diversity of rhizosphere bacterial communities between transgenic maize 2A-7 and its control. As shown in Figure 2, the richness estimator ACE and the diversity indices Simpson and Shannon were not significantly different between the transgenic maize 2A-7 and its control at the same developmental stages. These results suggest that the artificial modification of Bt proteins in maize does not affect the diversity of rhizosphere communities.

### 2.3. Beta Diversity of Rhizosphere Bacterial Community of the Two Maize Lines

**Figure 3:**
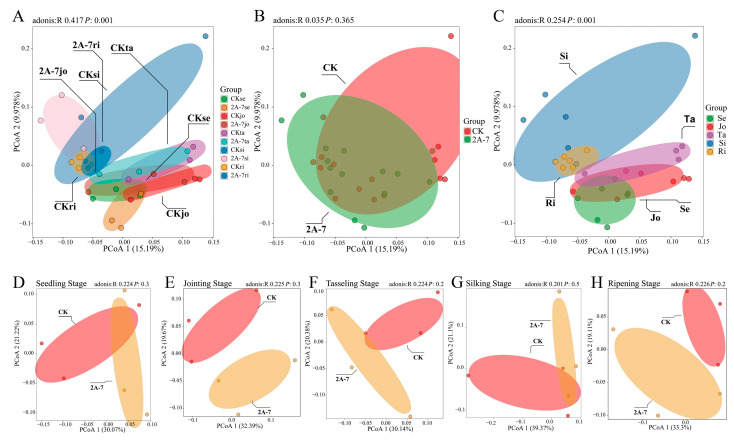
Principal co-ordinate analysis (PCoA) based on UniFrac distances of bacterial communities in the rhizospheres of transgenic maize 2A-7 and the control maize cultivar at different growth stages. (**A**) PCoA using two variables: different growth stages and maize inbred lines; (**B**,**C**) PCoA using one variable: different maize inbred lines (**B**) or different growth stages (**C**); (**D**–**H**) PCoA using different maize inbred lines as a variable at seedling stage (**D**), jointing stage (**E**), tasseling stage (**F**), silking stage (**G**) and ripening stage (**H**). The adonis R value represents the overall variation that can be explained by a certain grouping pattern in the PCoA analysis. The *p* value represents the significant difference based on Student’s *t*-test (*p* < 0.05). CK, non-transgenic control Dongdan 6531. Se—seedling stage; Jo—jointing stage; Ta—tasseling stage; Si—silking stage; Ri—ripening stage.

To further analyze the impact of transgenic maize 2A-7 on the bacterial communities in rhizosphere soil, a principal co-ordinate analysis (PCoA) was conducted. Using different growth stages and maize inbred lines as two variables, the analysis revealed that bacterial communities in the rhizosphere soils of different maize varieties at different growth stages were significantly different (*p* = 0.001) (Figure 3A). To explain the key factors causing these differences, we separately analyzed the impact of developmental periods and different maize varieties on the diversity of bacterial communities. The results showed that the difference in maize varieties did not cause significant change in bacterial communities (Figure 3B). However, when the developmental stage was taken as the only variable, significant differences were observed in the bacterial communities among different development stages (*p* = 0.001) (Figure 3C). Specifically, PCoA revealed that there was no significant difference in the beta diversity of bacterial communities in the rhizosphere soil of the two maize varieties during the five growth stages (Figure 3D–H). Therefore, we suggest that the main factor affecting the changes in the diversity of rhizosphere bacterial communities is the growth stage rather than the variety of maize.

### 2.4. Bacterial Community Composition

**Figure 4:**
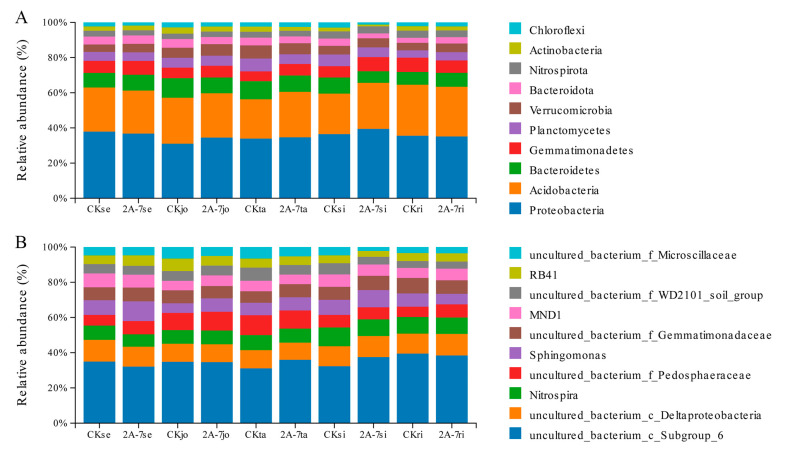
The composition of bacterial communities in the rhizosphere soils of transgenic maize 2A-7 and non-transgenic control Dongdan 6531. (**A**) The relative abundance of the top 10 phyla; (**B**) the relative abundance of the top 10 genera. CK, non-transgenic control Dongdan 6531. Se—seedling stage; Jo—jointing stage; Ta—tasseling stage; Si—silking stage; Ri—ripening stage.

The bacterial communities in the rhizosphere soil of transgenic maize 2A-7 and the control maize Dongdan 6531 are primarily clustered in 38 phyla, 93 classes, 186 orders, 272 families, 430 genera, and 528 species. The predominant bacteria at the phylum and genus levels were selected for further analysis based on a threshold of relative abundance > 1%. We found that Proteobacteria, Acidobacteria, Bacteroides, Gemmatimonadetes, Planctomycetes, and Verrucomicrobia were the dominant bacteria in the rhizosphere soils of the two maize varieties, accounting for over 80% of the relative bacterial abundance in total (Figure 4A). On the genus level, most of the bacteria could not be cultured; however, Nitrospira, Sphingomonas, MND1, and RB41 were the main known bacterial genera for the two maize varieties (Figure 4B). Overall, we found no significant difference in the bacterial community’s composition between the two maize species at either the phylum or genus level (Figure 4).

### 2.5. Co-occurrence Network Analysis of Maize Rhizosphere Microbiome

**Figure 5:**
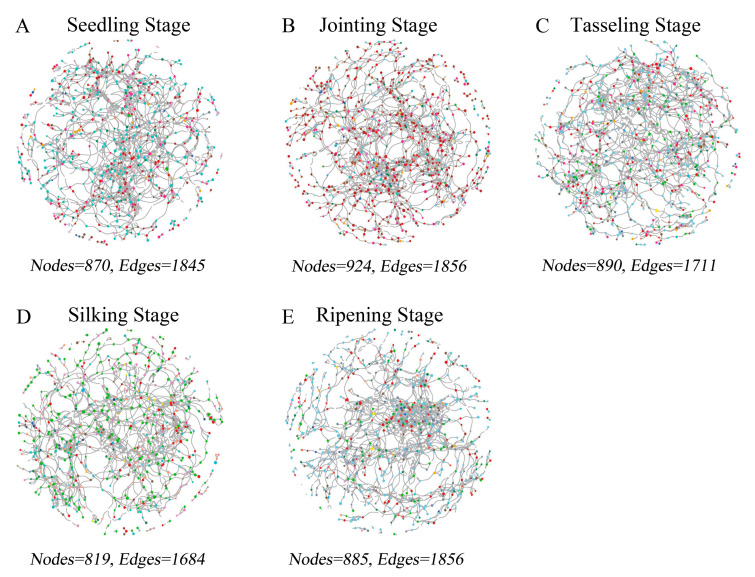
Networks of bacterial OTUs at different developmental stages in the two maize cultivars. OTUs in the same phylum are marked in the same color. (**A**) Seedling stage; (**B**) Jointing stage; (**C**) Tasseling stage; (**D**) Silking stage; (**E**) Ripening stage.

To analyze the impact of different developmental stages on bacterial communities in maize rhizosphere soil, we constructed five co-occurrence networks based on OTU data from different growth stages of the transgenic maize 2A-7 and its control Dongdan 6531 (Figure 5). In general, the complex of the rhizosphere bacterial communities was similar at different stages, as networks possessed similar numbers of nodes and edges (Figure 5). As OTUs belonging to the same phylum were marked with the same color, we could easily observe dynamic changes in the dominant bacteria in rhizosphere soils at different developmental stages (Figure 5). The co-occurrence was most stable at the jointing stage, as reflected by the largest number of nodes and edges, as well as minimum values for connectance, clustering coefficients, and degree centralization (Table 1). The microbiome network had the smallest diameter and betweenness centralization at the seedling stage, while the highest values for degree centralization were recorded at the silking and ripening stages (Table 1).

### 2.6. Linear Discriminate Analysis Effect Size (LEfSE) Analysis of Maize Rhizosphere Microbiome

**Figure 6:**
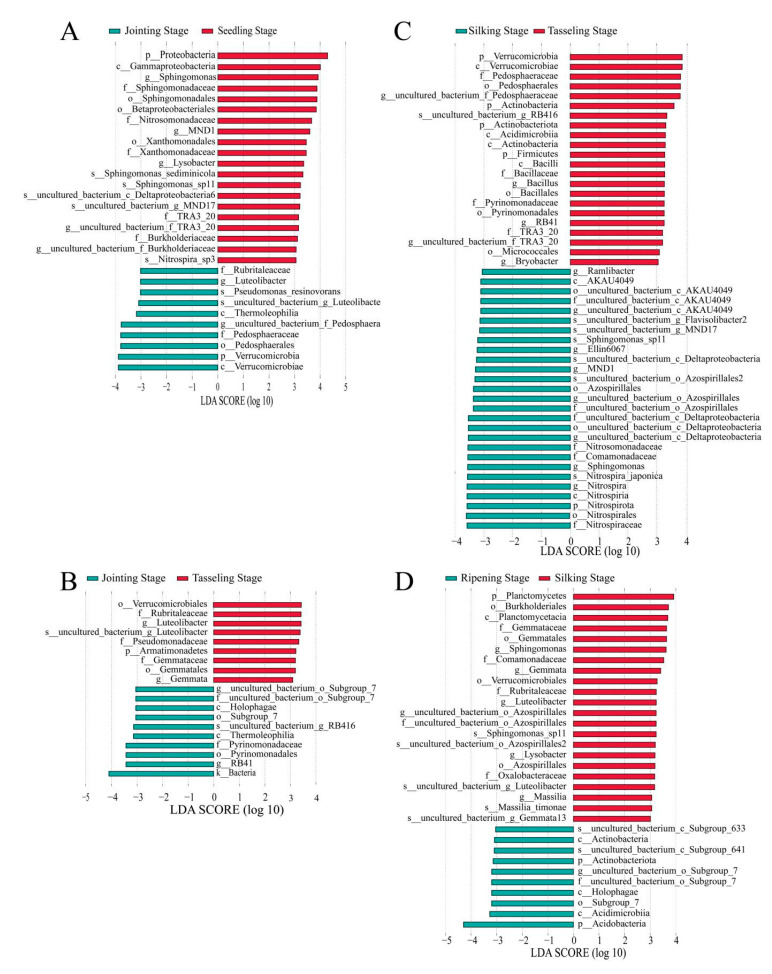
LDA scores of discriminative bacteria in the rhizosphere samples between two adjacent growth periods. (**A**) Jointing stage vs. seedling stage; (**B**) Tasseling stage vs. jointing stage; (**C**) Silking stage vs. tasseling stage; (**D**) Ripening stage vs. silking stage.

LEFSE analysis (LDA > 3) was conducted on the genus level to identify the bacterial biomarkers causing the composition changes in the rhizosphere bacteria of transgenic maize 2A-7 and its near-isogenic control Dongdan 6531 at different developmental stages. Comparing the jointing and seedling stages, we found that Sphingomonas and Lysobacter were the dominant bacteria in the seedling stage, while the genus Luteolibacter was significantly enriched at the jointing stage (Figure 6A). In the comparison between the tasseling and silking stages, the dominant bacteria belonged to the RB41, Gemmata and Luteolibacterde genera, respectively (Figure 6B). Comparing the tasseling and silking stages, we found that the dominant bacteria in the tasseling stage were of the RB41, Bacillus and Bryobacter genera, while bacteria belonging to the Ramlibacter, Elin 6067, MND1, Sphingomonas, and Neurospora genera were most abundant at the silking stage (Figure 6C). A comparison between the silking and ripening stages revealed that bacteria in the Sphingomonas, Gemmata, Luteolibacter, Lysobacter and Massilia genera were enriched at the silking stage, while the predominant bacteria at the ripening stage were Actinobacteria, Holophagae, and Acidimicrobiia (Figure 6D).

### 2.7. Redundancy Analysis (RDA) of Bacteria Community Diversity with the Environmental Factors

**Figure 7:**
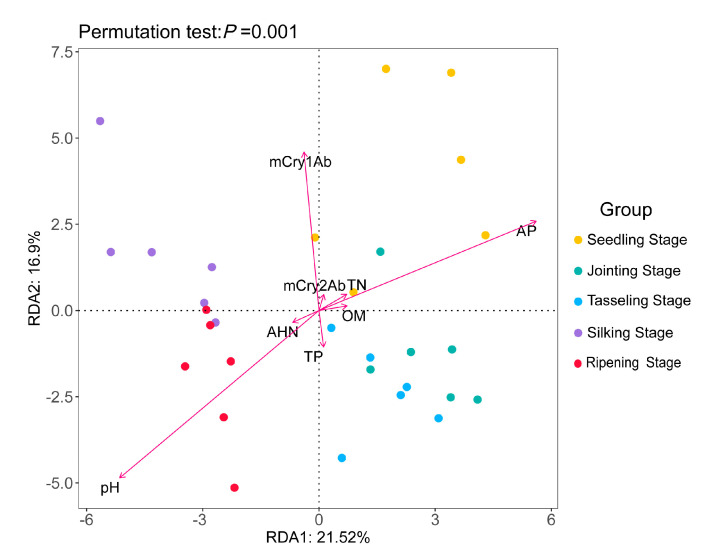
Redundancy analysis (RDA) of dynamic changes in bacteria communities in response to environmental factors. The arrows represent the environmental factors. TN—total nitrogen; AP—available phosphorus; TP—total phosphorus; AHN—alkaline hydrolyzable nitrogen; OM—organic matter. The length of the arrow line represents the degree of correlation; the longer the line, the greater the correlation.

To identify the relationships between the diversity of bacterial communities and other environmental factors, an RDA analysis was performed. The changes in two environmental factors, as well as the content levels of two modified Bt proteins and certain physicochemical properties [33], were used in this analysis. The results of the RDA analysis revealed that the pH level and available phosphorus content were the two most important environmental factors affecting the diversity of bacterial communities in the rhizosphere soils of the two maize varieties (Figure 7).

## 3. Discussion

Rhizosphere soil microorganisms, consisting mainly of bacteria, are microorganisms which closely attach to rhizosphere soil particles. The amounts of bacteria in rhizosphere soil are much higher than in bulk soil, sometimes ten or more times higher. Such microorganisms establish a balanced relationship that is interdependent and mutually reinforcing with the roots of their host plant. Rhizosphere microorganisms can convert inorganic substances into organic substances and secrete growth-stimulating factors, thereby providing sufficient nutrients for the growth and development of plants. In turn, the plant roots provide suitable conditions and sources of nutrients and energy for rhizosphere microorganisms [34,35]. The microbial communities of rhizosphere soils can be influenced by various factors, including species, genotype, pH, and others [36,37]. The genomes of transgenic crops contains exogenous insertion fragments capable of producing proteins from other species, and these proteins can be released into the soil through root exudates, pollen, and residues [38,39,40]. Different transgenic crops exhibit different accumulation patterns of exogenous proteins in the soil during different growth stages [8,41,42,43,44]. In this study, the two modified Bt proteins mCry1Ab and mCry2Ab were artificially synthesized by optimizing the codons of the original proteins Cry1Ab and Cry2Ab, respectively [45]. mCry1Ab contains three classic structural domains of Cry1Ab protein, which are closely related to its toxicity, but missing the C-terminal fragment with 531 amino acid residues. The mCry2Ab protein showed much higher similarity to the Cry2Ab protein (99.68%), which was obtained only through codon optimization at two specific sites [45]. The contents of the two modified Bt proteins in the rhizosphere soil of transgenic maize 2A-7 were detected at different development stages in the Summer of 2020. A slight difference between the change trends of the two Bt proteins was found, i.e., the content of mCry1Ab was highest in the rhizosphere soil at the seedling and silking stages, while the content of mCry2Ab remained relatively stable during all growth periods (Figure 1). This difference may be related to the characteristics (stability and water-solubility, etc.) of the two Bt proteins, and the soil conditions (type, pH, temperature, water content, and other soil physicochemical properties, etc.), as has been observed by other researchers [8,10,43,44].

As concerns over the ecological safety of GM crops continue to grow, more studies have been conducted on the impact of GM crops on soil ecosystems. Most studies have shown that changes in the microbial communities in rhizosphere soil are closely related to environmental factors and seasonal changes, but not to the planting of transgenic GM crops [18,28,30,39,46]. Compared with the effects of seasonal transition, the planting of Cry1Ac transgenic eggplant produces only a minor impact on soil bacterial communities [18]. The field residue of Cry3Bb transgenic corn MON863 has no impact on changes in bacterial communities in soil, but does have a small impact on fungal communities, mainly due to changes in environmental factors, which are not related to differences in the variety of corn [39]. In other studies, researchers have found that the planting of Cry1Ah transgenic maize HGK60 produces no significant differences in the composition and diversity of bacterial communities compared to controls at the same growth stage, but differences have been identified among different growth periods [30,47]. In the current study, a one-year field trial was adopted to investigate the impact of the transgenic maize 2A-7 on the composition and diversity of microbial communities. High-throughput 16S rDNA sequencing was used to compare the microbiomes of rhizosphere soils from transgenic maize 2A-7 and its control line. The alpha and beta diversity analyses of the bacterial communities in the rhizosphere soils of transgenic maize 2A-7 and its control lines revealed no significant differences between the two maize cultivars during the same growth periods (Figure 2 and Figure 3). However, samples at different developmental stages were distinct (Figure 3C). Therefore, developmental stages, rather than the cultivars, are the most important factor in determining the diversity of bacteria communities in rhizosphere soils. This finding is consistent with the results of most previous studies. Composition analysis at the phylum and genus level also revealed that the rhizosphere soils of the two maize cultivars in the current study had similar bacterial compositions at the same developmental stages (Figure 4), while soils at different stages had dominant bacterial species which were distinct (Figure 6).

Co-occurrence network analysis can reveal non-random associations between soil microorganisms. Ecological rules for microbial community structure and assembly have been gradually applied to the diversity analysis of various soil microorganisms [48]. Chen et al. found that with increases in pH value, the relationship between microbial communities in soil becomes closer [49]. Using co-occurrence analysis, Fang et al. found that microbial communities have a more complex and stable structure in summer compared with winter [50]. In the current study, the distribution patterns of bacteria in the rhizosphere soils of two maize cultivars at different stages were analyzed using a co-occurrence network analysis. It was found that the complexity of bacterial communities was similar at each developmental stage, but the centralization of bacterial communities was much higher at later developmental stages (Figure 5). Further LEFSE analysis showed that microbial community markers were different at adjacent developmental stages (Figure 6).

In a previous study, we found that there were no significant differences in the physicochemical properties and key soil enzyme activities of the rhizosphere soils of transgenic maize 2A-7 and its control line at the same developmental stage [33]. Compared to environmental factors, the growth period seemed to have the most significant impact on the bacterial communities, and materials from different growth periods distributed distinctly in different quadrants (Figure 7). In the present work, a redundancy analysis of dynamic changes in bacterial communities with environmental factors showed that changes in bacterial communities were significantly correlated with the pH value and the available phosphorus content (Figure 7). This finding is consistent with that of Li et al., who found that bacterial community composition was more closely correlated with soil pH values than with content levels of Bt protein [37]. In addition, we also found that mCry1Ab seemed to have a correlation with bacterial community changes (Figure 7). As mCry1Ab protein content has a strong positive correlation with the seedling and silking stages (Figure 1 and Figure 7), we speculate that the impact of mCry1Ab protein on the rhizosphere soil bacterial communities partially stems from the growth period. Taking into account other environmental and growth period factors, mCry1Ab’s impact on the soil bacterial community is not prominent; it is, at least, smaller than the impact of the growth period, pH and available phosphorus content.

Overall, we may state that there were no significant differences in the composition and diversity of bacterial communities in the rhizosphere soils of transgenic insect-resistant maize 2A-7 and its control Dongdan 6531 observed in the one-year field trial. The growth stage, rather than the Bt protein, is the main factor affecting changes in bacterial communities.

## 4. Materials and Methods

### 4.1. Plant and Soil Materials

Insect-resistant maize 2A-7 and the near-isogenic non-transgenic maize Dongdan 6531 were provided by China Agricultural University [33,45]. The two maize cultivars were planted in Jinan, Shandong, China (N 36°41′50″, E 117°04′33″) in the summer of 2020, using a randomized block design. Three replicates were given for each maize cultivar. The experimental site has cinnamon soil, which is neutral to weakly alkaline with middle level fertilizer. Seven days before planting, 3000 kg/acre of organic fertilizer and 30 kg/acre of compound fertilizer containing nitrogen (N), phosphorus (P), and potassium (K) were buried in the soil at a depth of 18 cm. During the entire growth period, no insecticides and herbicides were sprayed, but artificial weeding was carried out when necessary. Samples were collected from plant rhizosphere soils at five developmental stages, as described in our previous study [33], and then quickly transferred to liquid nitrogen prior to storage at −80 °C in a refrigerator in the laboratory.

### 4.2. Bt protein Content Determination

Cry1Ab was tested using the Cry1Ab/Cry1Ac protein ELISA kit (AP 003 CRBS, Envirologix, Portland, USA). Cry2Ab was tested using the Cry2A protein ELISA kit (AP 005 CRBS, Envirologix, Portland, USA). Standard curves were drawn based on the OD values of standard proteins at different concentrations to calculate the content of Bt proteins in each sample.

### 4.3. DNA Extraction and Sequencing

The DNA of the rhizosphere soil samples was extracted using a Soil Genomic DNA Extraction Kit (DP336, Tiangen, Beijing, China) following the manufacturer’s instructions. The DNA purity and concentration of all the samples was measured with a Qubit dsDNA HS Assay Kit and Qubit 4.0 Fluorometer (Thermo Fisher Scientific, Waltham, MA, USA).

The universal primers (27F: AGRGTTTGATYNTGGCTCAG and 1492R: TASGGHTACCTTGTTASGACTT) were used to amplify the full-length 16S rRNA gene from the genomic DNA of each sample. To meet the multiplex sequencing requirement, both the forward and reverse 16S primers were tailed with sample-specific PacBio barcode sequences. We used a KOD One^TM^ PCR Master Mix (KMM-101, Toyobo, Osaka, Japan) for PCR amplification with the following program: 2 min at 95 °C for initial denaturation, followed by 25 cycles of 10 s at 98 °C for denaturation, 30 s at 55 °C for annealing, and, finally, 2 min at 72 °C for extension. All the PCR productions were purified using Agencourt AMPure XP Beads (Beckman Coulter, Indianapolis, IN, USA) and quantified with a Qubit dsDNA HS Assay Kit and Qubit 4.0 Fluorometer (Thermo Fisher Scientific, Oregon, OR, USA). SMRTbell libraries were established from the amplified DNA with a SMRTbell Express Template Prep Kit 2.0, following the constructions of the manufacturer. Purified SMRTbell libraries were then sequenced on a single PacBio Sequel II system.

### 4.4. Bioinformatic Analysis

The BMK Cloud (Biomarker Technologies, Beijing, China) was used to carry out most of the bioinformatic analysis. Raw data were filtered and demultiplexed using the SMRT Link software with the minPredicted Accuracy ≥ 0.9 and the minPasses ≥ 5 as the criteria to obtain the circular consensus sequencing (CSS) reads. After low-quality reads were eliminated through filtering, trimming and duplication deletion, unique reads were obtained. Sequences with a similarity of ≥97% were clustered into one operational taxonomic unit (OTU). Based on the naive Bayes classifier in QIIME2 [51], taxonomy annotations of the OTUs were assigned with a confidence threshold of 70%. The alpha diversity was calculated and graphed using QIIME2 and R software, respectively. Based on the OTU table, the UniFrac distances among different samples were calculated. PCoA analysis was used to analyze the beta diversity using the R package vegan and ggplot2. The bacterial co-occurrence networks were constructed with the R packages igraph, psych, Hmisc and WGCNA (Spearman |ρ| > 0.7 and *p* < 0.01) based on the OTU matrixes of individual growth stages. Linear discriminate analysis effect size (LEfSe) with the PERMANOVA based on 9,99 permutations was then employed between two adjacent growth stages using a web tool (https://bioincloud.tech/standalone-task-ui/lefse, accessed on 16 May 2023) to find the biomarkers (LDA > 3). The redundancy analysis (RDA) was carried out using the R packages vegan and ggplot2 with the OTU table.

### 4.5. Statistical Analysis

All the statistical analyses were performed using the R software (version 4.1.0). Student’s *t*-test was used to calculate the significant differences between two groups based on *p*-values. A permutational multivariate analysis of variance (PERMANOVA) was used to examine significant differences among more than three groups. The LDA values in the linear discriminate analysis effect size (LEfSE) part were set to 3.0 to search the microbial biomarkers.

## Figures and Tables

**Table 1:** The parameters of the bacterial OTU networks at five different stages.

Period	Connectance	Average Degree	Average Path Length	Diameter	Clustering Coefficient	Betweenness Centralization	Degree Centralization
Seedling Stage	0.0049	4.24	9.61	23	0.43	0.053	0.014
Jointing Stage	0.0044	4.02	10.43	33	0.39	0.085	0.010
Tasseling Stage	0.0043	3.84	10.30	28	0.40	0.073	0.008
Silking Stage	0.0050	4.11	10.61	30	0.43	0.079	0.018
Ripening Stage	0.0047	4.19	11.00	39	0.41	0.080	0.016

## Data Availability

The Sequence Read Archive (SRA) accession number for 16S rDNA sequencing clean data is PRJNA943098. The data presented in this study are available in the graphs and tables provided in the manuscript.
